# Bioinspiration and Biomimetic Art in Robotic Grippers

**DOI:** 10.3390/mi14091772

**Published:** 2023-09-15

**Authors:** Van Pho Nguyen, Sunil Bohra Dhyan, Vu Mai, Boon Siew Han, Wai Tuck Chow

**Affiliations:** 1 School of Mechanical and Aerospace Engineering, Nanyang Technological University, Singapore 639798, Singapore or ngvphobk08@gmail.com (V.P.N.); sunilbohra.dhyan@ntu.edu.sg (S.B.D.); 2 Schaeffler Hub for Advanced Research at NTU, Singapore 637460, Singapore; hanbon@schaeffler.com; 3 Faculty of Engineering, Dong Nai Technology University, Bien Hoa City 76000, Vietnam; maithevu@dntu.edu.vn

**Keywords:** robotic gripper, soft gripper, conventional gripper, bioinspired gripper, biomimetic gripper, hybrid gripper

## Abstract

The autonomous manipulation of objects by robotic grippers has made significant strides in enhancing both human daily life and various industries. Within a brief span, a multitude of research endeavours and gripper designs have emerged, drawing inspiration primarily from biological mechanisms. It is within this context that our study takes centre stage, with the aim of conducting a meticulous review of bioinspired grippers. This exploration involved a nuanced classification framework encompassing a range of parameters, including operating principles, material compositions, actuation methods, design intricacies, fabrication techniques, and the multifaceted applications into which these grippers seamlessly integrate. Our comprehensive investigation unveiled gripper designs that brim with a depth of intricacy, rendering them indispensable across a spectrum of real-world scenarios. These bioinspired grippers with a predominant emphasis on animal-inspired solutions have become pivotal tools that not only mirror nature’s genius but also significantly enrich various domains through their versatility.

## 1. Introduction

Robotics has gained substantial popularity and holds the potential to facilitate a myriad of remarkable tasks [1,2,3]. Manipulation, which plays a pivotal role in the realm of robotics [4], constitutes an emerging and intricate field. In contemporary times, several materials, actuators, and fabrication techniques have been employed to develop grippers [5,6,7]. Within the landscape of prior research, robotic grippers can be broadly categorized into two principal groups: the traditional/conventional variety and soft grippers, both of which draw inspiration from natural mechanisms. Indeed, nature presents a compelling array of attributes that render it a rich source of emulation for the creation of continuously actuated structures. These qualities include optimized design, evolutionarily honed uniqueness tailored to specific tasks, and energy-efficient kinetics and dynamics to ensure survival [8].

This paper is dedicated to a comprehensive review and categorization of gripper designs derived from preceding works, wherein the design and actuation principles were inspired by natural mechanisms. The intent of this paper is to provide a contemporary overview of existing developments in the field of gripper technology, thereby offering valuable insights for fellow researchers. The paper encompasses three overarching themes: human-inspired, animal-inspired, and plant-inspired gripper concepts.

### 1.1. Grasping Taxonomy

**Figure 1:**
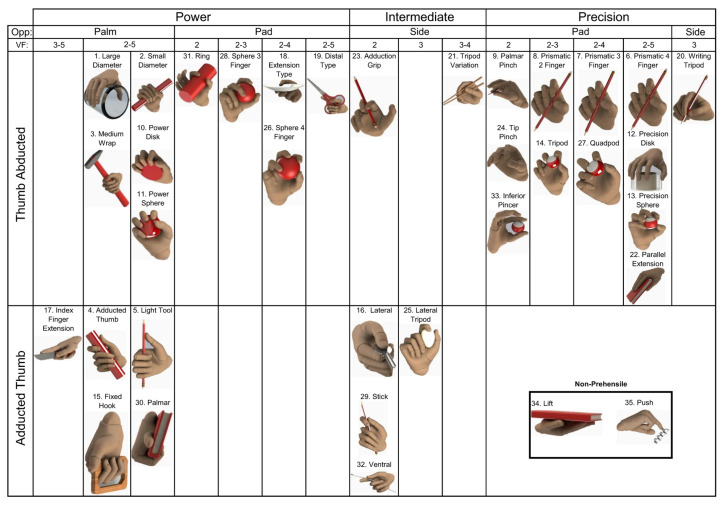
Grasping taxonomy of human hand based on the poses of the thumb, hand, and fingers [9] (© *2018, Scientific Data 5*).

Grasping, an intricate procedure involving the application of force from the fingers, palm, arm, and body to establish contact and secure a substrate, is often used to refer to the function of grippers. Depending on the physical and chemical properties of the substrates and the features of the grippers, there are many types of grasping. The human hand, distinguished by the multifarious DoF in each finger, and certain animal appendages demonstrate remarkable adaptability in accomplishing object manipulation. For instance, the authors of [9,10,11] summed up and categorized human grasping types into 32 types, as illustrated in Figure 1. According to this categorization, the human hand uses one or multiple fingers, fingertips, phalanges, and occasionally the palm, each tailored to specific cases. Presently, R-Hs equipped with five fingers, directly inspired by the human hand’s architecture, have made significant strides in emulating the aforementioned grasp types. These R-Hs exhibit designs rooted in traditional or hybrid structures, effectively mirroring the diverse spectrum of grasping methodologies elucidated earlier.

While human-inspired grippers are renowned for their widespread use across daily life and industries, offering impressive stability and agility, certain challenges persist due to limitations in their DoF. In contrast, the natural world presents a diverse array of mechanisms through which animals and plants seize or grasp objects in their surroundings. These mechanisms serve as blueprints for gripper designs. In addition to the common principles shared with human-inspired designs, animal- and plant-inspired grippers augment their grasp capabilities through microstructures present in their bodies, such as suction, wet adhesion, dry adhesion, and interlocking. Furthermore, when compared to their human-inspired counterparts, these grippers exhibit greater flexibility and reduced stability, owing to the vast and unrestricted DOF stemming from their anatomical structures.

### 1.2. Number of Objects

Grippers can be categorized into single-object grasping and multiple-object grasping based on how many objects they can handle in a single attempt. Single-object grasping [12] is a prevalent approach in manipulation, where the gripper focuses on manipulating one object during each cycle. Previous research on grippers and manipulation has predominantly concentrated on this single-object grasping concept, delving into aspects like mechanism design, grasp analysis, perception, and sensing. On the other hand, multiple-object grasping represents an innovative avenue in manipulation. This concept was first introduced in a theoretical model by the Harada team in 2000 [13]. Recently, in [14,15], researchers proposed a hybrid gripper equipped with two symmetrical arrays of fingers, each covered by soft pads, enabling it to handle multiple elongated objects. This hybrid gripper’s capabilities were further expanded to manipulate objects of varying shapes through the incorporation of cord supports within rigid skeletons [16,17]. In another effort, a group [18,19] commenced training the Robotiq gripper to detect and manage clusters of objects in a single trial. Sun [20] not only categorized the various types of multiple-object grasping but also developed perceptual enhancements for his gripper.

## 2. Human-Hand-like Grippers

The human hand consists of a combination of rigid bone phalanges and flexible ligamentous joints that make it the most advanced manipulator among all animals. The hybrid nature of the human hand contributed to the strength as well as dexterity of human fingers [21], which enabled them to handle intricate objects and construct complex structures. Due to differences in the shapes, sizes, and surface properties of the objects to be grasped, each case requires a specific grasping configuration. Schlesinger [22] categorized hand-grasping postures into six functional types based on the shape characteristics of both the hand and the grasped objects. These categories included fingertip pinch, side pinch, clamp pinch, hook, spherical grab, and cylindrical grab. Building upon this, Napier [23] highlighted the influence of the surface characteristics, size, and shape of the target object on grasping movements, leading to a distinction between precision grip and power grip.

Traditional robotic grippers are constructed with rigid joints and links, making them a prevalent choice for industrial robotic applications. These grippers operate using conventional transmission systems like gear-link transmission or tendon-driven mechanisms. They excel in structural stiffness and load-bearing capabilities, delivering substantial force for meticulous control and operation. As outlined in [24], these traditional grippers encompass a spectrum ranging from robot grippers with two or three fingers to multi-finger and adaptive grippers, depending on the finger count and adaptability techniques.

While traditional grippers offer agility in manipulating objects, they pose challenges due to mechanical and control intricacies. Particularly, they struggle with handling soft, deformable objects [4]. Moreover, their high structural stiffness renders human interaction unsafe [25]. To surmount these drawbacks, researchers have turned to soft robotic technologies, prioritizing end-effector compliance and adaptability for various objects [26,27]. The biological realm underscores the value of softness and compliance in simplifying interactions with the environment [28]. Soft grippers predominantly utilize SMPs, SMAs, and low-melting-point alloys (LMPAs) responsive to temperature changes. Additionally, stimuli like pH, light, electricity, and magnetism have been explored for advancing soft-compliant gripper development.

Despite substantial advancements in manipulator gripping research, attaining an equivalent degree of robustness and adaptability to that found in the human hand remains a challenging endeavour [29]. Existing R-Hs vary from simple single-DOF parallel grippers to high-DOF anthropomorphic hands. Some R-Hs utilize one or a few actuators with limited DOF, while others integrate fully actuated, passive joints and multiple DOF [30,31,32,33,34,35,36].

### 2.1. Traditional Rigid-Link Grippers

In this section, we conduct a comprehensive examination of traditional rigid-link grippers, encompassing a spectrum from simple two-fingered configurations to complex anthropomorphic hands featuring articulated fingers and joints. These grippers are activated through either external or integrated motors, utilizing contact-driven direct actuation or tendon-driven actuated structures.

#### 2.1.1. Two-Finger Grippers

**Figure 2:**
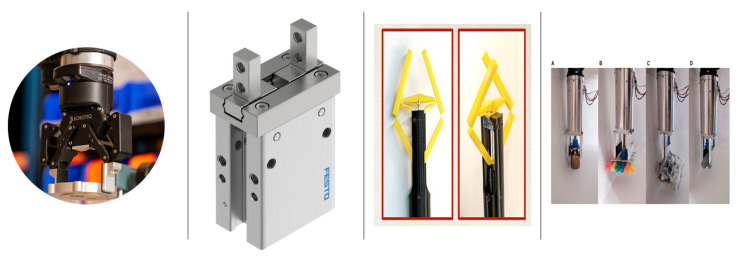
Some two-finger rigid-link grippers (from left to right): Robotiq two-finger gripper [37] (© *Robotiq Inc.*); Festo parallel gripper [38] (© *The Festo Group*); Two-finger gripper with roll and twist [45] (© *2022 Maxwell Samuels, Lu Lu, Cong Wang, reproduced with permission*); Versatile two-finger and retractable suction cup gripper [47] (© *2023 Frontiers*).

Two-finger robot grippers, also known as parallel claw grippers, represent a prevalent category of industrial grippers due to their straightforward usability, manufacturability, and cost-effectiveness. These grippers find extensive application in pick-and-place operations, palletizing, assembly tasks, and other uncomplicated manipulations. Prominent manufacturers in this domain include Robotiq [37] and Festo [38], known for producing well-regarded industrial grippers as shown in Figure 2.

Researchers have dedicated significant efforts to advancing two-finger grippers in order to enhance object manipulation, taking inspiration from the way humans predominantly utilize two fingers—the index finger and thumb—for in-hand manipulation [39,40,41]. To extend the capabilities of two-finger robots, several authors, including those in [42,43,44], introduced additional DOF by implementing actuated moving surfaces on the fingertips. These solutions, however, generally confine gripper motion to a single plane and limit in-hand manipulation to rolling motions without the ability to twist.

Maxwell [45] pioneered a multi-DOF two-finger robot gripper designed to handle larger objects and execute in-hand rolling and twisting (refer to Figure 2). Employing a tendon-driven mechanism to operate the linkages, this gripper also incorporates a folding mechanism, enabling it to function effectively in tight spaces. This particular gripper has proven valuable for conducting minimally invasive surgeries in humans.

Zhiguo [46] developed a two-finger dexterous bionic hand featuring three distinct grasping patterns: power grasping, precision pinch, and lateral pinch. This bionic hand boasts six DOF, with the thumb and index finger each composed of three rigid phalanx links and three rotational joints.

In another advancement, researchers in [47] introduced a versatile gripper that seamlessly blends two of the most prominent gripping technologies: two-finger and suction-cup grippers (refer to Figure 2). This innovative design involves a retractable rod-mounted suction cup located within the R-H’s palm, carefully positioned to avoid interference from the two fingers. By utilizing a single actuator to drive both finger motion and the sliding rod, this gripper minimizes complexity while achieving versatile functionality.

#### 2.1.2. Three-Finger Grippers

Three-finger grippers, as the name suggests, are composed of three fingers utilized for grasping objects. While these grippers are more intricate compared to their two-finger counterparts, they find application in scenarios demanding heightened precision and accuracy for handling delicate items.

The BarrettHand™ BH8-282 [48], developed by the University of Pennsylvania, is a notable example of a three-finger gripper. It features three articulated fingers and a palm, enabling object grasping. With a high degree of flexibility through its eight axes, this gripper, controlled by four brushless DC servomotors, can adapt in real time to securely encompass diverse objects. Each finger is independently controlled by servomotors, operating two joint axes. The Torque Switch™ facilitates torque transmission through joints, aiding in the grasping process. While the inner and outer fingers follow an anthropomorphic design, the opposing thumb’s motion replicates non-anthropomorphic primates’ spreading motion. Precise control over position, velocity, acceleration, and torque is achievable for each finger across 200,000 encoder positions. Additionally, integrating tactile and strain-gauge sensors enhances BarrettHand’s grasp intelligence.

In the realm of three-finger grippers, the success of grasping is reliant on the interaction between object and hand strategies. In the context of underactuated hands, Model S [49] introduced a novel approach. Consisting of three coupled prismatic joints and single-joint fingers, this 3D-printed ABS hand adapts passively to an object’s principal axis. By curling all three fingers around an object until they tangentially make contact with its curvature, this grasp maximizes contact and minimizes dropping risks [50]. Similarly, Schunk’s three-finger R-H uses a single common actuator to drive three prismatic joints. The works of Hanafusa and Asada [51] present a radially symmetric three-finger hand featuring rollers at the fingertip to reduce tangential friction during grasping. The MLab hand [52] incorporates elastic elements into its radially symmetric fingers, enhancing conformance to grasped objects. Vignesh [53] introduced an underactuated gripper with three fingers driven by linkage and six DOF, leveraging unconventional anthropomorphic designs and 3D-printed PLA plastic fabrication. Incorporating a spring mechanism for compliance, a single motor powered the fingers through a gear train mechanism, providing differential motion. This design process drew on online human grasp data for parameterization and validation through 3D-printed models.

#### 2.1.3. Multi-Finger Grippers

Multi-finger and adaptive grippers bring the ability for robots to adeptly conform to objects of varying shapes and sizes, enhancing grip strength and overall reliability. One of the early instances of such a gripper was the UTAH/MIT Dexterous Hand [54], a robotic end-effector jointly developed by the University of Utah and the Massachusetts Institute of Technology. Created as a research tool for comprehending machine dexterity, the Dexterous Hand (DH) emulated anthropomorphic design principles, inspired by the versatile capabilities of the human hand in performing intricate manipulation tasks. With over twenty-five DOF, including its wrist, the DH prioritized speed and strength to interact with materials spanning from soft foams to rigid metals. To enable this, the actuation system was positioned external to the hand for enhanced flexibility, and flexible tensile tendons were employed to transmit energy from actuators to the hand. The configuration of thirty-two individual actuators controlled the tendons, which boasted a lifespan of six million cycles [55,56].

The Stanford-JPL hand [57] introduced a distinct approach by siting motors on the forearm, with flexible conduits housing Teflon-coated tension cables that looped around the wrist joints. This strategic arrangement reduced the impact of gravity and inertia from hand actuators on the entire system. In doing so, the design minimized the hand’s dimensions and improved its durability by eliminating the need for actuators within the fingers.

The realm of multi-fingered R-Hs also encompasses the JPL four-fingered hand [58] and the Anth R-H [59]. The latter, Anthrobot, stands out with its focus on achieving anatomical similarity to the human hand, encompassing not only the finger count but also aspects such as thumb positioning, movement, link lengths, and palm shape. While the hand consists of a total of 20 joints (four per finger and four for the thumb), it leverages 16 motors for control.

The robot hands previously mentioned are governed by remote actuation systems, utilizing flexible tendon cables. However, these cables introduce inaccuracies in joint angle control, and the intricate mechanical design can complicate maintenance. In response, researchers directed their efforts toward crafting robot hands featuring integrated actuation systems within the finger bodies and palms.

Examples of such internally actuated hands include the Belgrade/USC hand [60], the Omni hand [61], the NTU hand [62], and DLR’s hand [63]. A notable instance is the DLR/HIT Hand I, developed by Liu [64], comprising four fingers with a total of thirteen DOF. The subsequent iteration, DLR/HIT Hand II [65], a five-fingered hand with fifteen DOF, was designed to resemble the human hand more closely while integrating the actuation and communication systems within the fingers’ bases. Each finger is composed of a finger body housing super-flat BLDC motors and a finger base accommodating a bevel gear transmission, facilitating curling and extension motions. However, the last two joints of the fingers are mechanically coupled and controlled by steel wires, preventing the individual control of the middle and distal phalanges. The thumb, not utilized in the DLR/HIT Hand I, was fixed appropriately in the DLR/HIT Hand II design, allowing all five fingers to be conveniently positioned on the palm using spring probes [64].

Another noteworthy development is the Gifu Hand II [66], an anthropomorphic robot hand featuring a thumb and four fingers. The thumb comprises four joints with four DOF, while each finger boasts three DOF across four joints. Similar to the DLR/HIT Hand II, built-in servo motors are integrated into all finger joints for easy palm adaptor mounting. Notably, the Gifu Hand II employs a distributed tactile sensing system with 624 sensing points, coupled with a six-axis force sensor in each finger. This configuration enables compliant pinching and squeezing. The incorporation of high-stiffness gears, such as satellite gears, instead of tendon-driven harmonic drive gears contributes to a high-stiffness hand.

### 2.2. Soft Grippers

In this section, we undertake an extensive exploration of soft grippers, spanning from pliable two-fingered grippers to intricate, heavily compliant anthropomorphic hands with articulated fingers and joints [67,68,69]. These grippers exhibit various activation methods for grasping objects, including gripping by actuation, gripping by controlled stiffness, and gripping by controlled adhesion.

#### 2.2.1. Soft Grippers with Gripping by Actuation

Grippers employing external actuation utilize servo-motors positioned outside the gripper body to drive the mechanism and achieve object conformation. In this category, numerous soft-compliant grippers have emerged, characterized by a grasping action accomplished through a singular actuator at the gripper’s base, facilitating the bending and buckling of the structure [70,71,72,73].

Petkovic devised a two-finger underactuated compliant gripper exhibiting concave and convex grasping shapes. Distributed compliance defined its operation, and the entire gripper was 3D-printed as a single unit, thus ensuring cost-effectiveness and adaptability to diverse objects [71]. In a similar vein, Bruno [72] integrated proprioceptive haptic feedback into a two-finger self-adaptive gripper. A novel sea-saw mechanism enabled object grasping without external vision systems. The gripper’s stiffness determined the point of contact with the object. Further advancing this field, Liu [73] conceived a two-finger adaptive compliant gripper (ACG) synthesized through a soft-add topology optimization algorithm. This involved a holistic size optimization approach, amalgamating the augmented Lagrange multiplier (ALM) method, simplex method, and nonlinear finite element analysis. In the study, three distinct silicon rubber prototypes were fabricated. The ACG tool showcased proficiency in manipulating items like clips, coins, batteries, forks, and USB drives.

Furthermore, researchers have also ventured into the development of tendon-driven underactuated grippers, drawing inspiration from the human hand’s mechanics. These grippers employ a single tendon to drive multiple DOF, mirroring the dexterity of the human hand. Generally, such grippers utilizing tendon-driven actuation are composed of rigid links and joints [36,74]. Here, our focus is on compliant soft grippers driven by tendons. Raymond [75] spearheaded the Yale OpenHand Project, dedicated to creating commercially viable underactuated hands. The T42 model featured two autonomously driven, opposing underactuated fingers, constructed via 3D printing. This innovation enabled the gripper to handle objects ranging from delicate glass cups to staplers, showcasing its versatility. Ali [76] introduced a B-I-compliant 3D-printed soft gripper boasting sensorless compliance. This design proved adept at manipulating fragile items like strawberries and even intricately detailed 3D-printed artificial ears. Such advancements in tendon-driven underactuated soft grippers offer promising avenues for enhanced object manipulation across a wide spectrum of tasks and objects.

Manti [77] introduced a B-I three-finger soft-robotic gripper designed for adaptable and effective object grasping. This gripper employed a single actuator within an underactuated C-D mechanism. Similar to the human hand, each finger was comprised of three phalanges. The flexion and extension of these fingers were accomplished through cables that traversed a U-tube channel within each finger. These cables were anchored to spools and connected to the motor shaft at the gripper’s base. The gripper’s variable stiffness enhanced its ability to grasp various objects, including cylinders, spheres, plastic boxes, eggs, fresh tomatoes, and compact discs. Luke [78] proposed a caging-inspired gripper employing three hooked flexible fingers and a mobile palm configuration, forming a cage-like structure around the object while gripping. The flexible fingers, behaving like pre-loaded springs, offered both conformity to object shapes and increased friction for objects not suited for caging. This gripper demonstrated efficient grasping for cuboid and cylindrical objects, making it suitable for supermarket applications, particularly in handling fruits and vegetables.

Additionally, researchers in [79] presented a lightweight tendon-driven robotic gripper with three fingers and a telescopic palm, actuated pneumatically. These fingers exhibited swift pre-contact movements and could grasp and release objects—such as Starbucks cups, plastic bottles, and baseballs—at high speeds. Ahmed [80] developed a pneumatically actuated gripper incorporating rubber hose fingers with a nonuniform wall thickness. This design was tailored for handling small, lightweight, and delicate objects like electronic chips and light bulbs. Each finger was activated by controlled air pressure, enabling the precise gripping and manipulation of objects with the utmost care.

The SDM Hand [81] stands out as a robust four-fingered compliant gripper designed for unstructured environments. Fabricated using polymer-based shape deposition manufacturing, this hand integrates actuators and sensors into rigid polymers. The gripper operates using a single actuator to drive all four fingers, simplifying the mechanism and reducing its weight. Notably, each link’s concave side features a soft finger pad to optimize the friction and contact area, thereby enhancing the gripper’s ability to grasp [82,83]. The SDM Hand has exhibited its capabilities by manipulating objects such as a volleyball, wooden block, wine glass, wine bottle, and box of matches. Xu and Todorov [84] took a similar approach, developing an anthropomorphic hand capable of mimicking human hand behaviours and performing various tasks. Likewise, the PISA/IIT SoftHand [27] represents an innovative robot hand with 19 joints, leveraging a single actuator to activate adaptive soft synergies. The key innovation of this hand lay in its use of inventive articulation and elastic ligaments, replacing the conventional rigid links and joints typically found in R-Hs. This design choice significantly enhanced the hand’s dexterity and adaptability.

#### 2.2.2. Soft Grippers with Gripping by Controlled Stiffness

Soft grippers that utilize shape-memory alloys or polymers react to thermal stimuli, causing them to change their shape and conform to objects of varying sizes and shapes [85,86]. Lan [87] introduced a self-sensing two-finger microgripper made of an SMA, capable of handling objects like strings, screws, and even a fly. Each finger incorporated resistance for the self-sensing of the finger’s position.

**Figure 3:**
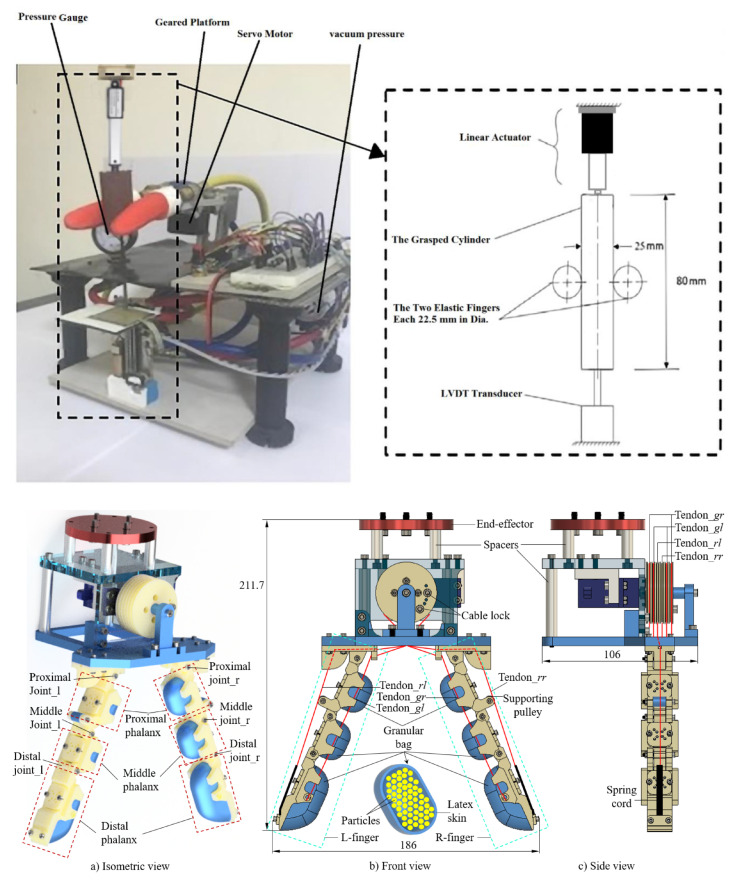
Soft grippers with controlled stiffness: (**top**) two-finger R-H granular jamming gripper [90] (© *MDPI Electronics Volume 12 Issue 8*); (**bottom**) two-finger granular tendon R-H [91] (© *MDPI Micromachines Volume 14 Issue 7*).

Control-stiffness-based grippers operate on a principle distinct from that of the actuation-based grippers mentioned earlier. These grippers initially reconfigure their structural composition to become soft and then envelop the object to be grasped without causing harm. Researchers have explored several variable-stiffness technologies, including granular jamming, low-melting-point alloys, and shape-memory materials [77,88]. Granular jamming grippers function by manipulating loose particles within a bag, depressurizing them to create a solid object or introducing air to make the gripper soft. While ground coffee is a common choice for jamming particles, glass, plastic, and metallic beads have also been used [46]. Lipson and Amend proposed a two-finger R-H with granular jamming fingers, demonstrating high dexterity in manipulating small objects such as chopsticks [89]. Similarly, Osamah [90] developed a two-finger R-H that utilized vacuum pressure to control stiffness and the finger structure to prevent slippage during grasping as seen in Figure 3 (top). Chow introduced a two-finger granular tendon gripper as shown in Figure 3 (bottom), with a hybrid structure composed of a rigid finger-like skeleton and attached granular pouches [91]. This gripper could lift various objects, including cylinders, brackets, sachets, pulley gears, and bolts.

Low-melting-point alloys (LMPAs) are materials that undergo a phase transition from solid to liquid when exposed to heat. This property is harnessed to achieve variable stiffness structures in the fingers of grippers [92]. Shintake [93] developed a two-finger soft gripper that integrated an LMPA track and pre-stretched dielectric elastomer actuator (DEA). The LMPA track within the finger responded to thermal energy. In its liquid state, the gripper exhibited a soft structure, and electrostatic actuation from the DEAs flattened the finger. To achieve rigid finger shapes, heat was removed from the LMPA while keeping the DEAs actuated. Despite weighing only 2 g, the gripper was able to lift a plastic dish containing 11 grams of metal washers, which was 5.5 times its own weight. SMPs and SMAs have also been employed to adjust the stiffness of grippers through phase transitions. Given that SMPs exhibit greater relative stiffness changes and lower moduli in both rigid and soft states compared to SMAs, our focus is primarily on using SMPs coupled with soft actuators as variable-stiffness components [94,95,96].

Researchers Patel [97] and Thrasher [98] developed three-fingered grippers made from UV-curable elastomers. These grippers offered a modular approach to manipulation tasks. Grippers based on SMPs and SMAs have also been explored for their ability to provide variable stiffness. Wang and Anh [99] introduced an SMP-based soft gripper with three identical fingers, each featuring a soft-composite actuator with a changeable-stiffness material. The fingers incorporated embedded Ni-chrome wires for heating the SMP structure, transitioning it from a glossy state to a rubbery state. The gripper could successfully grasp various objects, such as an egg, a capsicum, a hollow cylinder, and pyramid-shaped objects.

Origami-based tendon-driven three-fingered grippers (often referred to as robogami) were proposed by Firouzeh and Paik [100,101,102]. These grippers were manufactured using layer-by-layer techniques and integrated SMPs to control joint stiffness. The grippers adapted themselves to different objects using quasi-2D fabrication methods. Experimental tests included power and precision grasps, involving the manipulation of an egg with a power grasp and a coin with a precision grasp.

Mingfang [103] presented another shape-memory-alloy-based soft gripper with variable stiffness, featuring an 18-fold stiffness enhancement for each finger. This gripper’s core design included a self-activated actuator with flexible bending deformation and shape-retaining abilities, incorporating two changeable-stiffness joints. The gripper’s construction involved five types of materials: SMA wire, paraffin, Ni-Cr wire, silicone rubber, and PLA plastic. The gripper’s variable stiffness allowed it to pick up objects such as a square box using actuated joint 2 and a plastic cup or an orange using actuated joint 1.

Since SMAs and SMPs exhibit changes in stiffness through phase transitions, they have been utilized by researchers to develop multi-finger grippers. Behl [104] presented a four-finger gripper with a bi-directional SMP that was able to pick up and place a coin. Moreover, She [105] developed an R-H where each finger was comprised of SMA material strips. The integration of SMA materials into grippers allows for greater flexibility in object manipulation. In addition, Shahinpoor [106] developed a four-finger gripper using ionic polymer metal composites (IPMCs).

#### 2.2.3. Soft Grippers with Gripping by Controlled Adhesion

The fluidic elastomer actuator (FEA) is one of the oldest actuation technologies in soft robotics and was used by Suzumori [107] to develop a four-fingered gripper. The gripper consisted of three pneumatic chambers that allowed for the dexterous grasping of objects like a beaker containing liquid and a metallic wrench, as well as the ability to tighten a bolt without the need for external tools. Several other researchers have developed multi-finger grippers or R-Hs using FEAs, including a six-fingered gripper [108] that could bend upwards and downwards and pick up a raw egg, a five-fingered R-H presented by Yamaguchi [109] that was able to pick up a cup and tape roll, and an R-H introduced by Niiyama [110] with pneumatic pouch actuators and structures that could be printed in 15 min.

## 3. Animal-Inspired Grippers

In this section, we delve into the art of animal-inspired grippers, exploring their diverse forms through the lens of eight major taxonomic categories: clamp, suction, wrapping, dry adhesion, wet adhesion, swallow, lock, and hook. Each of these taxonomic groups encompasses a spectrum of gripping mechanisms, often with additional subdivisions capturing even finer nuances. Certain grippers blend elements from multiple taxonomic groups, resulting in hybrid designs that draw inspiration from various aspects of the animal kingdom.

### 3.1. Clamp Grasp

**Figure 4:**
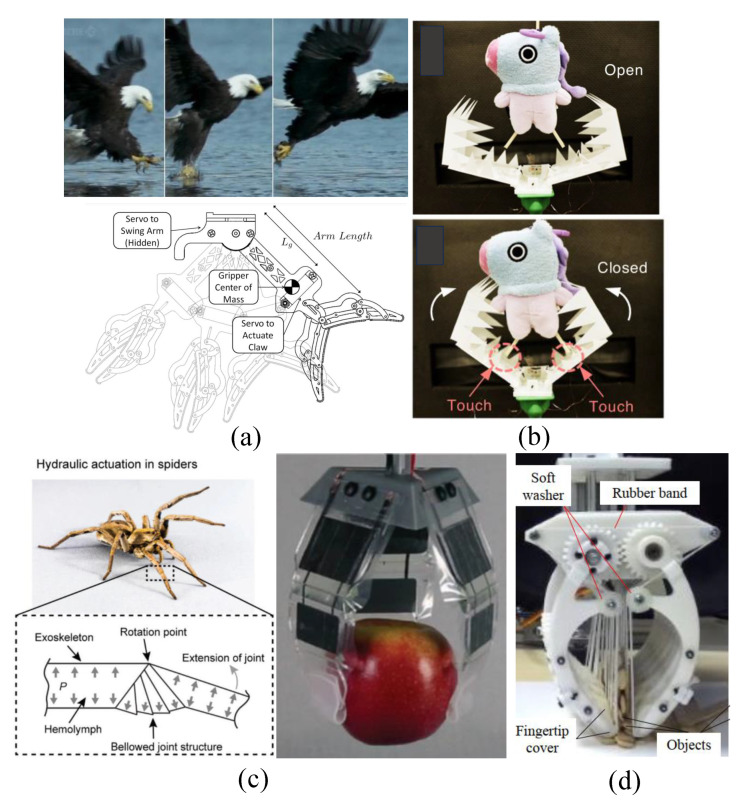
B-I grippers based on clamping mechanism mimicking different animals: (**a**) bird (avian) [112]; (**b**) origami [119]; (**c**) spider [121]; and (**d**) crab [17] (© *IEEE Robotics and Automation Letters*).

Gripping objects through the application of squeezing forces, generated by anatomical features such as fingers and legs, represents one of the most prevalent methods in the animal kingdom. This mechanism has also found popularity in the field of robotic gripper fabrication, where it has been widely imitated. An example of this is the CCRobot-II, a gripper inspired by monkeys, as described by Zhenliang et al. [111]. This gripper is equipped with a palm-based cable system that enables it to traverse bridges. Featuring dual palms—one on each side—it generates the required squeezing force through passive springs and gear transmission to maintain its grip on the bridge structure. Predatory birds possess the remarkable ability to swiftly capture prey with high success rates across diverse conditions. Justin et al. [112] proposed a gripper inspired by avian digits, integrated onto a quadrotor UAV to achieve rapid object grasping (refer to Figure 4a). Comprising two opposing fingers, this gripper employs a DC servo motor for gripping and releasing actions. Other similar gripper mechanisms have been developed, often incorporating enhancements for more gentle interactions.

As an illustration, the work presented in [113] showcased the design of a soft-clamp gripper featuring two opposing Pneunet fingers actuated by pneumatic pressure. These fingers were constructed from a series of chambers made from silicon rubber, featuring a cross-sectional area that gradually decreased from the palm to the tip. This specific morphological configuration empowered the gripper to adeptly manipulate objects with shapes such as spheres, cylinders, blocks, and discs. A separate instance is represented by the jamming fingers introduced in [90], which incorporated two latex membranes containing volumes of small particles. The gripper’s motion and stiffness were regulated by controlling the air pressure through a tube system that connected the fingers to a pump. Moreover, a three-linkage gripper, outlined in [114], emerged from the collaboration of two rigid fingers, each equipped with three phalanges and operating with a single DoF. Drawing inspiration from the eel jaw, the gripper presented in [115] comprised two rigid fingers, each possessing three linkages. This configuration ensured that the fingertips maintained parallel movement in relation to each other, enhancing their grasping capability. Additionally, the studies conducted in [116,117,118] further contributed to the field of B-I grippers, particularly focusing on designs inspired by bird perching mechanisms.

Crabs have evolved a unique gripping mechanism involving multiple-linkage fingers organized into two claws, which has inspired advancements in gripper design. An example of this concept can be found in the work of Wenzhong et al. [119], who introduced an origami-based structure featuring two symmetrical fingers equipped with multiple wedges. These fingers exhibited the ability to open and fold through origami joints, with the wedges establishing contact between the fingers and the objects being manipulated (refer to Figure 4b). Further contributions to this area include the design and optimization of a crab-like gripper presented in the study by Linh et al. [120]. This gripper encompassed multiple rigid links in a compact form, offering both a miniaturized scale and compliant performance. Building upon these concepts, recent efforts described in our prior works [14,17] have introduced novel grippers characterized by two symmetrical claws attached to a central palm. Each of these claws incorporates an array of multiple fingers operating in synchronization via connecting shafts. Additionally, the hybrid structure of each finger integrates a cord stretched by a rigid skeleton. Demonstrating remarkable efficacy, these grippers have exhibited commendable performance in grasping various objects during trials (see Figure 4d). Figure 4c depicts a spider-like gripper with three fingers, actuated by electrohydraulic actuators, designed for swift manipulation [121]. This configuration involves one finger featuring three rigid phalanges capable of rotational movement around each other via a rotational joint actuated by the electrohydraulic mechanism.

The principle of a fin ray, integral for fish swimming and underwater balance, has also inspired the development of certain grippers. Notable examples of such grippers, borrowing from the fin ray effect, can be found in works like [122,123,124,125]. These studies introduced grippers incorporating finger structures and mechanisms resembling the fin ray’s operation. In these cases, the fin-ray-inspired fingers shared analogous structures and designs, characterized by a soft skin supported by multiple cross-beams mounted in parallel. This arrangement empowered the grippers to readily adapt to the contours of objects, particularly those with spherical shapes.

### 3.2. Sucking Grasp

The octopus, a remarkable eight-armed mollusk, showcases a distinctive array of arms, each adorned with numerous suckers on its mouth’s surface [126,127,128,129]. The cross-sectional profile of each arm is broader at its proximal base and gently tapers towards its distal tip. These arms, in conjunction with the suction cups, offer multi-functionality, serving roles in manipulation, locomotion, and chemotaxis perception. The octopus employs its arms for various behaviors, including crawling, walking, signaling, destructive camouflage, and prey extraction. This versatility has motivated the integration of octopus-arm-inspired concepts in manipulation research.

During the initial stages, an octopus-like arm was designed, composed of three segments equipped with two pneumatic muscles [130,131,132], controllable through air pressure. All muscles were stabilized using a twisted nylon wire. A similar approach was adopted in [133], featuring an arm with four segments, each comprising three pneumatic muscles. In [134,135,136], two octopus-inspired arms were manufactured, measuring 210 mm in length, 16 mm in diameter at the tip, and 21 mm in diameter at the body. These arms, made of silicon rubber, employed cable or SMA actuation, showcasing the ability to adapt to and lift substantial loads. Another soft octopus arm, featuring eight tentacles, utilized fluidic bending actuators to drive arm motion [137]. Jie [138] devised a flexible octopus gripper comprising two soft Pneunet fingers for gripping objects like apples.

**Figure 5:**
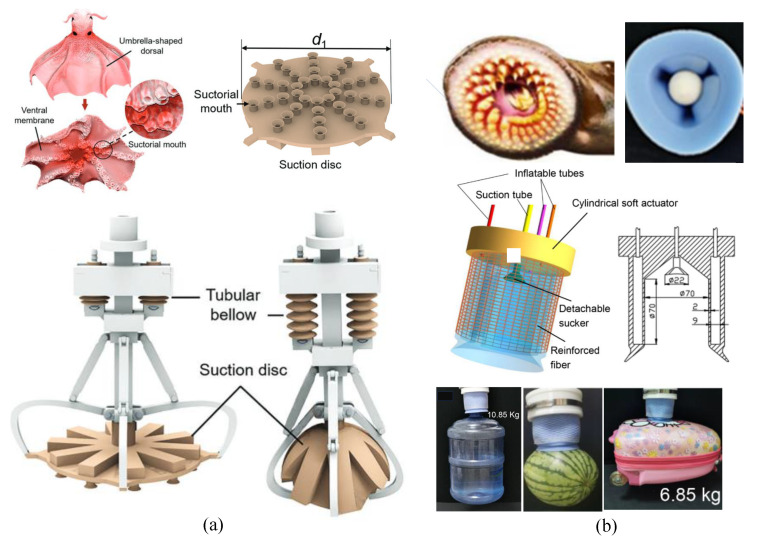
B-I grippers based on sucking mechanism mimicking different animals: (**a**) octopus [142] (© *Advanced Science, vol 9*) and (**b**) lamprey [143] (© *Actuators, vol 11*).

Recent developments have focused on enhancing suction cup functionality in octopus arms. For instance, the Barbara group [139] devised suckers with varying morphologies, enabling inner membrane movement via pneumatic means. Additionally, the suction force was improved through alterations in the taper angle and bending curvature of the suction cup [140]. Microscale suckers with PDMS structures were fabricated to enhance wet adhesion [141]. For improved self-adaptation underwater, Mingxin [142] introduced fins between adjacent arms and embedded light within each suction cup (see Figure 5a).

Another intriguing example is the lamprey-mouth-inspired gripper with concentrically mounted suction cups of varying sizes [143] (see Figure 5b). This innovative gripper exploited the suction force to pick up large objects exclusively. In contrast, the lamprey-mouth-like gripper in [144] employed a single suction cup, gripping objects using passive pressure within the cup chamber without external pneumatic assistance. An origami-based suction cup mechanism, inspired by birds, was introduced in [145].

### 3.3. Wrapping Grasp

Grippers equipped with multiple-link fingers embody a conventional design frequently found in industrial applications and production lines. While achieving gross movements with fine motor control might be straightforward [146], the presence of rigid links inherently limits the number of DoF available for the gripper. This limitation becomes pronounced when handling intricate or pliable tasks. In response, the concept of continuum robot grippers has emerged as a promising avenue, offering versatility across various industries [147,148,149,150]. These grippers excel in scenarios involving slender objects [151] and medical applications [152,153].

Continuum robots draw inspiration from living structures observed in the animal kingdom, exemplified by five primary mechanisms: the elephant trunk, serpentine motion, tentacle movement, octopus arm dynamics, and the flexible spines of mammals. This B-I foundation fuels their innovation, enabling the development of grippers capable of complex and adaptable operations.

#### 3.3.1. Elephant Trunk

The elephant trunk, often referred to as the proboscis, stands out as an extraordinary organ characterized by an exceptional range of movement, effectively functioning as a muscular hydrostat. This remarkable anatomical feature serves to not only manipulate objects of varying weights but also carry out a multitude of social and sensory tasks [154]. The absence of a rigid, articulated skeleton is compensated for by the coordinated contractions of antagonistic muscles, resulting in bending, twisting, elongation, shortening, and stiffness adjustments. The elephant trunk showcases remarkable versatility in its capabilities, allowing it to transport substantial loads of up to 270 kg while also engaging in intricate tasks such as delicately handling a single blade of grass. Beyond its physical strength, the proboscis serves as a multi-functional tool in an elephant’s daily life. These functions encompass not only breathing but also olfaction, mechanosensation, activities like siphoning or spraying water, vocalization, posture-based communication, tool utilization, and even sprinkling dust for various purposes [155,156,157,158].

**Figure 6:**
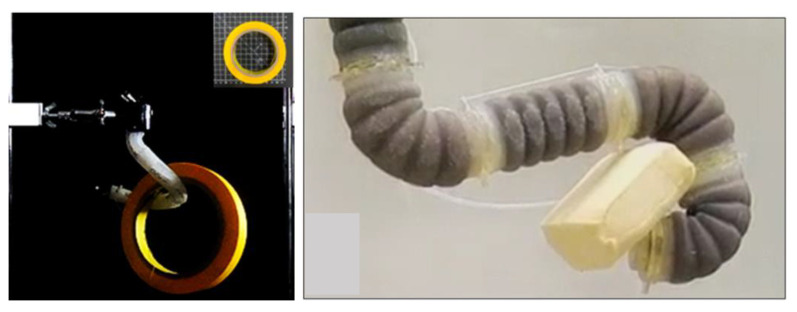
B-I grippers based on sucking mechanism mimicking the elephant trunk [160] (© *MDPI Polymers Volume 15 Issue 5*).

Wilson and his colleagues [159] can be recognized as pioneers in establishing the groundwork for the development of a robot inspired by the mechanics of an elephant trunk. In their innovative design, the soft robot was composed of a complete arm, culminating in a gripper affixed to its end-effector. Spanning a total length of 614 mm, this robot was created using various sections constructed from orthotropic and polyurethane tubes, employing a structural arrangement akin to the Pneunet concept. As a result of this arrangement, the gripper and arm exhibited bending or releasing motions in synchronization, mirroring the behaviors exhibited by an actual elephant trunk, whether under pressurized or nonpressurized conditions. A gripper inspired by the elephant trunk is also shown in Figure 6, where a SMA [160] was used to replace the C-D system in previous elephant trunk-like grippers to boost the flexibility of the gripper. Although the SMA made the gripper more compact in controlling multiple sections, a big challenge to achieving high accuracy was still present due to the heating and cooling-down process.

In a different approach, the authors of [161] developed a soft R-H that closely emulated the functionality of an elephant trunk, utilizing a C-D system. This hand was constructed with five interconnected segments, forming a chain-like structure. The external surface of each segment was composed of a soft tube made from a latex membrane sealed by two end caps. To enhance the segment’s stability, an inner space containing a spring and various sizes of jamming granular material was incorporated. Further refinements were introduced in subsequent designs [162,163], where a central backbone composed of a chain of shorter links interconnected by universal (cardan) joints was added to each segment. Moreover, a spring system was integrated to connect neighboring links and restrict their lateral motion. Advancements in this direction were made by Huang [164] and Zhang [165], who transformed the concepts from [162,163] into parallel-segment structures driven by cable systems. They subsequently developed mathematical models to systematically investigate, analyze, and assess the performance of these grippers in manipulation tasks.

#### 3.3.2. Tentacle-Inspired Grippers

Tentacles, observed in certain animal species like squids and snails, are remarkable appendages characterized by their mobility, flexibility, and elongation. Often found in invertebrates, these tentacles can occur in one or multiple pairs within an animal’s anatomy. Anatomically, tentacles typically function similarly to muscle hydrostats. These versatile structures serve a variety of purposes, primarily centered around feeding and grasping activities. Additionally, many tentacles function as sensory organs, exhibiting varying levels of sensitivity to touch, visual stimuli, and olfactory or gustatory cues, aiding in detecting specific food sources or potential threats. The diverse functionalities of these tentacles have inspired the development of robotic gripper designs that seek to emulate their unique capabilities.

In a squid-inspired design presented by Wilson et al. [159], a flexible gripper resembling a squid grasping a shrimp was proposed. This gripper featured two symmetrical Pneunet fingers controlled by air pressure on both sides. Grasping and releasing actions were achieved by adjusting the air pressure to intake or exhaust states, respectively. Each finger demonstrated side load and gripping force capacities of 1.42 N and 10 N, respectively. Harvard researchers introduced a tentacle-inspired gripper in [166]. This gripper consisted of spaghetti-filament-shaped fingers, each actuated by pneumatic pressure. The R-H incorporated 12 filaments arranged in concentric circles with 25 mm and 50 mm diameters. External fluidic control was facilitated by a pneumatic manifold connected to the filaments, enabling the gripper to adapt to and grasp various objects like balls, plants, and frames. Martinez’s group [167] developed a tentacle-like gripper using a composite elastomer system. This system utilized micropneumatic networks distributed across the different interfaces of two distinct elastomers, enabling intricate 3D motions. Other tentacle-like gripper designs were explored using origami-based [168] or C-D mechanisms [169]. Biomimetic suckers inspired by a squid’s adhesive capabilities, following the discovery that suckers removed from deceased squid could securely adhere to smooth surfaces [170], also influenced the design of squid-inspired grippers. Hou’s work [171] enhanced the gripping force of suckers in a squid-inspired gripper by incorporating silicon tubes. Mimicking jellyfish tentacles [172,173,174,175,176] and sea anemone [124] also inspired the development of soft grippers that can function underwater. The motions and grasping postures of tentacle-like grippers in object manipulation have been modeled and analyzed in various studies [177,178,179,180,181,182,183,184,185].

#### 3.3.3. Serpentes-Inspired and Mammal-Inspired (Spine-like) Grippers

**Figure 7:**
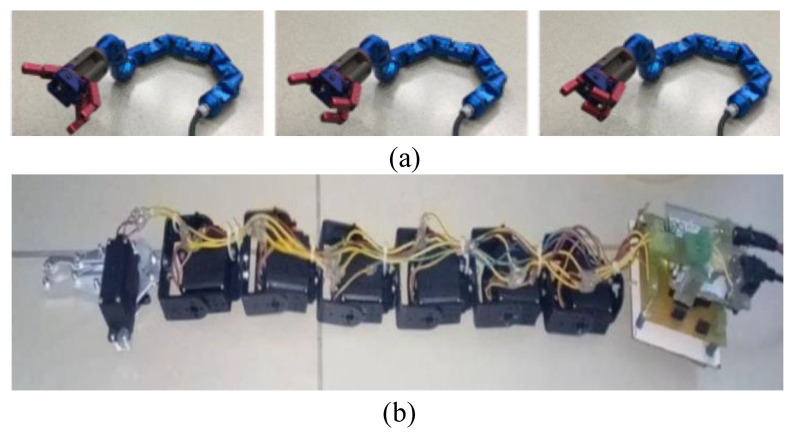
B-I grippers mimicking snakes: (**a**) [196] and (**b**) [199]. In such scenarios, the gripper modules are attached to the snake bodies.

The suborder Serpentes, commonly known as snakes, represents a group of reptiles characterized by their elongated limbless bodies, as discussed in [186,187,188]. Snakes achieve locomotion through the propagation of wave-like motions along their bodies. This unique locomotion mechanism allows them to move swiftly even in confined spaces, inspiring the design of robotic grippers [189,190,191,192,193,194]. A robot tongue design in [195] featured a metal-tape backbone enclosed in a PET tube, actuated by pneumatic pressure. A snake-like robot was introduced in [196] for exploration and rescue missions in narrow spaces. This robot gripper comprised three fingers, each with two phalanges, offering a total of 8 DOF. The gripper’s movement was facilitated by a gear transmission system. Additionally, the robot’s body could navigate through confined areas due to interconnected rigid segments with universal joints. Equipped with micro-cameras on its fingertips, this R-H could observe its surroundings without requiring the snake’s body to move (see Figure 7a). A fiber-tip microgripper, detailed in [197], employed a three-hinge mechanism and a spring system for passive actuation by squeezing. This microgripper was mounted on the head of a snake-like robot body for picking up thin paper. Other snake-inspired robots described by Kumar [198,199] incorporated multiple interconnected links driven by separate DC motors. These robots featured grippers with two fingers, directly actuated by a motor (see Figure 7b). The authors of [200] developed a helical-fabric robot hand with variable stiffness and contact-sensing capabilities. This gripper could alter its morphology by twisting and adapting to an object’s surface using hydraulic actuation.

Drawing inspiration from the human spine, a continuum actuator was employed for flexible manipulation, as demonstrated in [201]. This approach utilized dorsal vertebrae connected by SMA wires to form an arm. In a similar vein, the giraffe’s long and flexible neck, allowing it to manipulate objects at a distance, inspired the design of a continuum gripper presented in [202]. This gripper featured interconnected dorsal vertebrae stabilized by ligament systems, driven by pneumatic muscles. Experimental results showcased its ability to handle sizeable objects, such as a cup with a 250 mm diameter. Taking cues from monkeys that use their tails to hold onto branches, a hybrid soft robot gripper with three fingers actuated by pneumatic muscles was developed, as described in [203]. In a different approach, a spiral robot arm proposed in [204] employed a chain of multiple elements interconnected by an elastic backbone and actuated using a mutual-cable system. This arm demonstrated effective wrapping around objects like pens and rocks in three different versions, featuring one, two, or three driven cables.

### 3.4. Dry Adhesion Grasping

**Figure 8:**
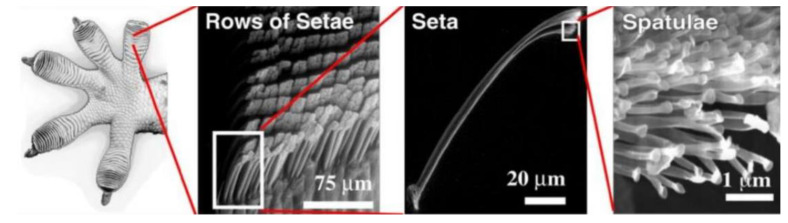
B-I grippers mimicking the toe sole structure of the gecko microstructure of a gecko toe sole [210] (© *MDPI Robotics Volume 11 Issue 6*), and gripper application [211] (© *Science Robotics Volume 6 Issue 61*).

Due to differences in evolutionary histories, the dermatoglyphics of animal toes or fingers are very diverse [205]. For instance, gecko feet feature apical ridges on their volar surfaces, which enable them to achieve dry adhesion to various surfaces in their environment through Van der Waals interactions among molecules or even single atoms [206,207]. The anatomical characteristics of these features facilitate efficient attachment between an animal’s toes and its surroundings. Consequently, researchers have extensively explored and leveraged this mechanism, as evident in both scientific investigations [208] and applications in robotics [209,210], particularly within gripper designs (refer to Figure 8).

As an illustration, a group of researchers in [212] developed a gripping mechanism utilizing thin silicon films covered with microscale wedges, serving as the surface of contact between the gripper and the objects. These films were affixed to Pneunet fingers, and when the air pressure within the finger chambers increased, the fingers bent towards each other, enabling a grasping motion. This approach combined dry adhesion from the wedges with the gripping force exerted by the fingers. Similar methodologies were explored in [211,213,214,215,216], where C-D two-finger robot hands were devised. In these studies, adhesive pads were divided into smaller scales and affixed to the phalanges of the fingers. The shear force, enhanced through electrostatic stimulation, played a pivotal role in securely grasping objects. Another noteworthy example is the robot hand presented in [217], featuring gecko-like adhesive pads on its fingertips for handling flat-surfaced objects. Each pad was actuated by a linear wire made from an SMA. Using 3D printing techniques, researchers in [218,219] fabricated a novel gripper with adhesive pads on its fingertips, designed to grasp flexible circuits or balloons. In another approach, an adhesive pad consisting of a three-layer thermoplastic polyurethane (TPU) material was developed in [220], demonstrating switchable adhesion on rough surfaces. This TPU adhesive pad exhibited successful performance in picking up circular or cylindrical objects, such as eggs and cans, by adjusting the supplied voltage.

In a recent development, a gecko-inspired gripper with switchable adhesive properties, driven by a magnetic field, was presented in [221]. This gripper featured a gecko-like surface composed of setal arrays and lamellar structures. The lamellar structures were designed to control dry adhesion through deformation induced by the magnetic field. Simultaneously, the setal array enhanced dry adhesion and contributed to self-cleanability at the contact interface. Another approach to modulating dry adhesion was demonstrated by Tian [222], who achieved this by altering the air pressure within the finger chamber system of the gripper. In the pursuit of optimizing gripping without exerting excessive force on objects, gecko-inspired grippers have been developed to address this concern. For instance, the gripper devised by Hawkes [223] featured two soft bands with gecko-like wedges covering the grasping interface. Experimental evaluations showcased the gripper’s ability to pick up and lift various objects with minimal applied force. This achievement was primarily attributed to the dry adhesive force generated by the gecko-like surface. Furthermore, the design of the finger skeletons aimed at generating minimal squeezing force, enabling the soft bands to effectively adhere to object surfaces.

### 3.5. Wet Adhesion Grasping

**Figure 9:**
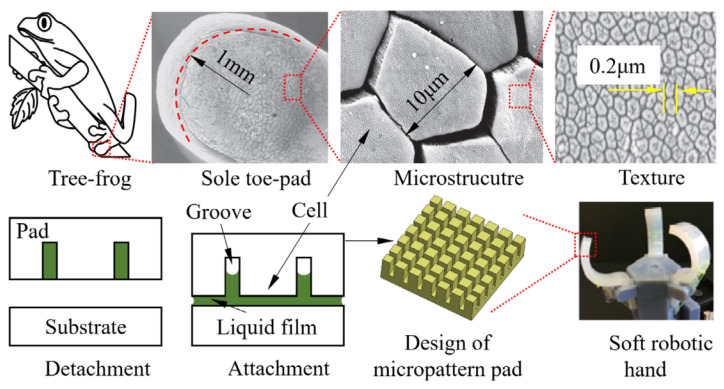
Tree-frog inspired gripper for handling soft and fragile objects under wet environments, such as contact lenses and food ([241] © *Bioinspiration & Biomimetics, vol 14*). In this scenario, the soft pads include a large number of blocks spaced by a network channel for boosting the wet adhesion force between the fingers and the object.

Similar to grasshoppers, tree frogs employ a remarkable mechanism to achieve adhesion, utilizing the principles of surface tension and capillary bridges [224,225]. Tree frogs excrete a liquid film into the gap between their toe pad sole and the substrate to stabilize their attachment. The sole surface of the toe pad is comprised of a multitude of hexagonal cells arranged in an array. Each cell also referred to as a block, has an approximately 10 µm diameter inscribed circle and is separated from neighboring cells by channels (grooves) with a depth of around 10 µm (refer to Figure 9). Before making contact with the substrate, most of the fluid remains within these channels to prevent rapid evaporation. Upon contact with the substrate, the liquid is rapidly expelled from the grooves, filling the gaps between the toe pad and the substrate. This mechanism generates the wet adhesion force that enables the toe sole to stick to the substrate. Consequently, tree frogs can adeptly interact with their surroundings under both wet and dry conditions. The grooves in the toe pad serve as reservoirs for generating and releasing the wet adhesion force between the toe pad and the substrate.

Utilizing wet adhesion by emulating the adhesion mechanism of tree frog toe pads offers a suitable approach for designing a soft R-H capable of autonomously grasping wet and deformable objects with minimal applied force. Previous researchers have extensively explored the wet adhesion principle by simulating how tree frog toes attach to substrates [226,227,228,229]. The investigation of hybrid surface patterns involving hexagonal micro-pillars constructed from various materials has been a focus of study [230,231,232,233]. These studies involve direct experimental comparisons to assess the effectiveness of different surface designs. The potential of tree frog-inspired grippers has been demonstrated in various works [234,235,236,237]. Additionally, investigations have been conducted into the role of patterned surfaces in enhancing contact forces between the tips of soft fingers, contributing to the understanding of effective design strategies [238,239]. This body of research collectively indicates that the emulation of tree frog-inspired wet adhesion has the potential to enhance the performance of soft robotic grippers, enabling them to adeptly handle wet and deformable objects while minimizing the force required for gripping.

In our prior research [240,241,242], we initiated a preliminary assessment of the wet contact between two surfaces: a micropatterned pad and a substrate (as depicted in Figure 9). Building upon this model, we subsequently applied it to grasp a delicate thin shell within a moist setting [243,244,245] with camera systems for detecting the contact, as well as a tofu block under dry conditions [246]. The evolution of tree-frog-inspired grippers has rendered them particularly suitable for manipulating fragile and soft objects within damp environments, all the while minimizing the necessity for exerting substantial squeezing forces prior to grasping.

### 3.6. Lock and Hook Grasping

The remora, also known as the suckerfish, belongs to the Echeneidae family [247]. Renowned for its distinctive ability to attach itself to a variety of marine creatures, the remora achieves this feat using an adhesive disc formed by its flexible and adjustable dorsal fin. This specialized mechanism enables the suckerfish to hitch rides on other aquatic organisms, irrespective of the fluid shear during high-speed swimming. In a study by Yueping et al. [248], a biologically inspired prototype was developed based on the morphology of the remora disc. The fabricated disc, measuring 127 mm in length and 72 mm in width and weighing 129 g, featured a soft lip encompassing eleven aligned rows of overlapping lamellae. Each row comprised numerous rigid carbon-fiber spinules, each measuring 270 μm, tapering to a sharp tip and arranged in a linear fashion. Experimental testing involving attachment to shark skin demonstrated that the fabricated disc generated frictional forces 34 times greater than the calculated drag force of 0.16 N in natural circumstances. This estimation was derived from a 35 cm suckerfish adhering to the rough skin of a shortfin mako shark, which was moving at an average swimming velocity of 1.5 m/s. Given the remarkable adhesive strength achieved by the disc, this research outcome holds significant potential for application in gripping objects within wet environments.

**Figure 10:**
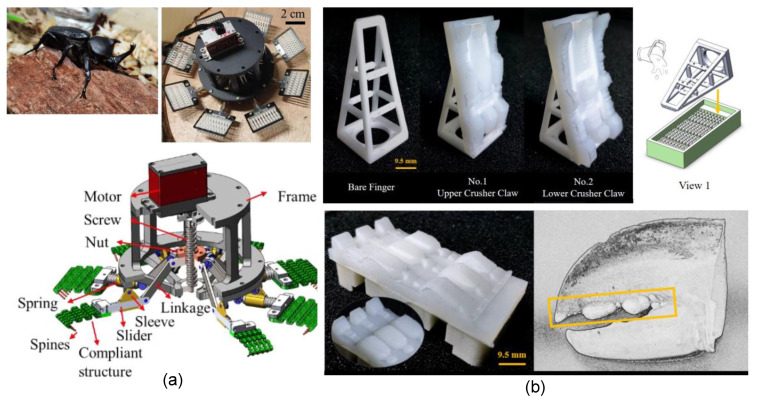
B-I grippers based on hooking and locking mechanism mimicking different animals: (**a**) beetle-leg-inspired gripper [250] (© *MDPI Biomimetics Volume 8 Issue 1*), and (**b**) lobster-jaw-inspired gripper [254] (© *2021 Frontiers*).

Furthermore, the concepts of lock and hook mechanisms have been applied to design grippers with characteristics similar to bees [249], beetles [250] (see Figure 10a), and ants [251,252,253]. These grippers feature fingers equipped with small hooks, allowing them to attach to surfaces. In another instance, a lobster-inspired gripper was introduced in [254], where the contact interface of each finger incorporated multiple sharp pins, enabling an interlocking mechanism when handling hefty objects (see Figure 10b).

### 3.7. Swallowing Grasp

**Figure 11:**
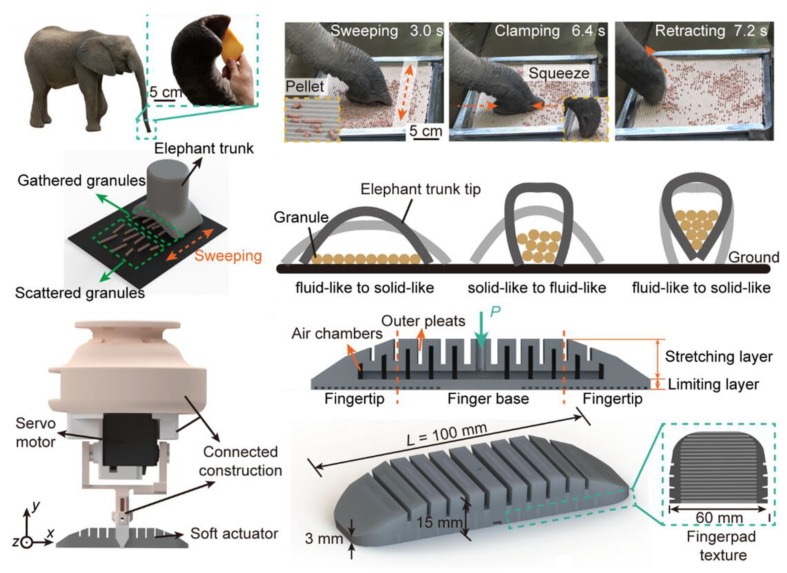
Swallowing-inspired grippers based on animal features such as an elephant’s nose [257] (© *MDPI Biomimetics Volume 8 Issue 4*).

Swallowing, scientifically termed deglutition, holds a crucial role in the consumption of food and liquids. It represents a physiological process in the lives of animals, during which sustenance or fluids traverse from the mouth down the pharynx and oesophagus while the epiglottis closes to prevent the entry of substances into the airway. More recently, the intricate process of swallowing has been investigated and emulated in the realm of robotic applications. For example, Dongbao and colleagues [255] undertook an analysis of the feeding mechanism employed by blood worms and subsequently developed an R-H that drew inspiration from this process for versatile gripping. This gripper design encompassed the ability to envelop the entirety of an object and furthermore achieved self-adaptive grasping with objects of varying sizes through the integration of detachable modules. Hong Bin in [256] proposed a bionic gripper inspired by the eating activity of an anemone, which had a similar principle to that in [255]. In addition to its rolling motion for manipulation, the elephant trunk possesses the capability to grip and pinch objects using its nasal appendage and digits. A couple of studies, namely [257,258], introduced a gripper design that emulated both the nose and fingers of the elephant trunk (refer to Figure 11). This gripper took the form of a suction cup, wherein the structure incorporated two symmetrical Pneunet fingers situated within a jamming membrane. Positioned at the center of the gripper was a hole that imitated the elephant trunk’s nasal portion. Consequently, the manipulation of air pressure within this gripper engendered three distinct mechanisms: the activation of the fingers, the jamming membrane, and the suction cup. Each of these mechanisms served a specific purpose—gripping, pinching, and suction—enabling a versatile approach to object manipulation.

The chameleon’s unique ability to swiftly extend its tongue, grab prey from varying directions, and elongate with remarkable flexibility has inspired researchers to develop novel soft grippers with similar functionalities. In [259], a gripper design was introduced that emulated the chameleon’s tongue mechanism, enabling the gripper to grasp objects at its tip. This gripper incorporated a tubular actuator, enclosed by a soft tongue skeleton functioning as its inner core and featuring a hole at the tip to mimic the chameleon’s tongue tip. Upon applying positive air pressure, the gripper’s tongue tip closed, thereby capturing the object. Another chameleon-inspired gripper concept was presented by Dong Jun in [260]. This design employed a rigid tongue structure, capable of rapidly shooting out and retracting for manipulation tasks. The gripper’s tongue achieved swift linear motion through the utilization of a wind-up spring and gear transmission, thus replicating the chameleon’s dynamic tongue movements.

## 4. Plant-Inspired Grippers

**Figure 12:**
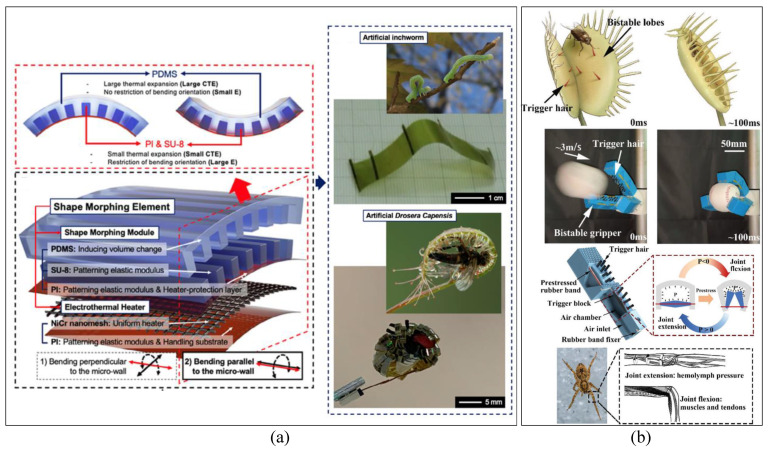
Mechanism of the shape morphing module based on the variation of the elastic modulus (E) and coefficients of thermal expansion (CTE) for different materials and the bioinspired showcases: the movements of the inchworm and Drosera Capensis (**a**) [261] (© *Advanced Intelligent Systems Volume 5 Issue 3*); a high-speed gripper imitating the fast predation process of the Venus flytrap, with the B-I design of the gripper including the repeatable trigger mechanism originating from the spider’s joint structure (**b**) [262] (© *Advanced Science Volume 8 Issue 21*).

Yongrok’s team in [261] developed soft grippers featuring shape-morphing films (SMFs) actuated through electro-heating (as depicted in Figure 12a). This research showcased the gripper’s versatility through three distinct grasping modes inspired by flower mechanisms, specifically Bauhinia variegata and Drosera Capensis, which utilize hierarchical morphology for insect trapping, as well as the inchworm locomotion pattern. The Venus flytrap’s ability to rapidly capture prey, especially insects, due to its responsive terminal lobes served as inspiration for another gripper design in [262] (see Figure 12b). This gripper was designed to excel in high-speed dynamic picking tasks. The gripper’s design incorporated two fundamental principles: snap-through instability that facilitated quick responses and a pneumatic control system influenced by spider mechanisms. As a result, this gripper was capable of successfully executing dynamic tasks such as capturing a swiftly moving baseball.

**Figure 13:**
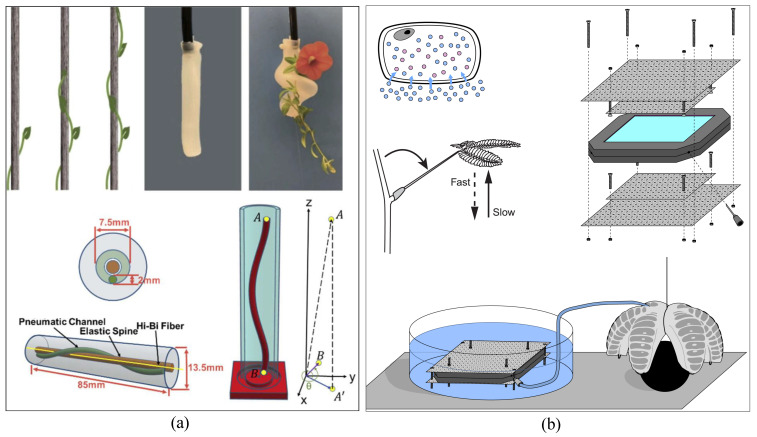
The twining mechanism of a twining plant on its substrate was imitated by the design of a soft gripper for handling a flower (**a**) [263] (© *Optics Express Volume 28 Issue 23*). A robotic gripper was actuated by an osmotic pressure generator inspired by the plant mechanism (**b**) [267] (© *PLoS ONE 2014*).

In [263], a soft spiral gripper was developed to imitate the twining mechanism observed in plants. This gripper utilized a single pneumatic control mechanism to secure the target object. Notably, it featured a fiber-optic sensor capable of providing information about the touch status and twining angles (as shown in Figure 13a). Experimental results indicated its effectiveness in handling objects with a maximum twining angle of 540 degrees, as well as its ability to firmly grasp even small objects with diameters of around 1 mm.

The vine-inspired gripper described in [264,265] was conceptualized based on a concentric spring-tube backbone that was operated using a cable system. This design incorporated a core composed of three sections, with each section containing a concentric carbon-fiber tube (as shown in Figure 13b). The tip and middle sections were equipped with a loaded-spring mechanism, allowing for the local extension and contraction of the gripper. To control the bending motions of the tubes, three cables were affixed to each tip section, enabling the gripper to achieve two DOF in bending each tube. Additionally, the gripper was fitted with a camera at its tip, which served to capture data during tasks involving remote inspection or object manipulation.

The unique hierarchical vein patterns found in cabbage leaves, which lead to their curling formation, served as inspiration for a bionic gripper design in [266]. This gripper featured two leaf-like structures, each of which was fabricated using 3D printing technology with a polylactic acid (PLA) polymer. These printed structures mimicked leaf veins and provided support to the lightweight leaf bodies, which were constructed using paper substrates. The gripper’s mechanism for grasping objects was based on controlling the heat generated by a PI electric sheet that was attached to the back of each leaf. By manipulating the temperature, the gripper could induce the curling behavior, effectively allowing it to grasp and hold objects. Another example of plant-inspired gripper technology is the osmotic pressure generator [267], which drew inspiration from plant mechanisms. This generator was integrated into a five-finger Pneunet gripper design. The operation of the generator was based on the concept of hydrated dialysis cassettes, utilizing water and a diluted liquid solution. By controlling the movement of the liquid solution in and out of the finger chambers through a pipe system and pressure transducer, the gripper could generate pressures of up to 5 kPa, enabling the effective grasping and manipulation of objects (as depicted in Figure 13b).

## 5. Summary

The B-I grippers discussed in the previous section showcase a diverse range of designs involving mechanisms, actuators, materials, and applications, all realized through various fabrication methods. This section aims to summarize the common features of these B-I grippers and generalize their key concepts.

### 5.1. Actuators

In almost all grippers, actuators were used to generate the motions for the fingers, and in a few cases to drive the palm. These actuators varied widely and were either used individually or in combination within each gripper design. From a technical perspective, these actuators can be categorized into the following major groups.

Direct-drive: In this configuration, a single motor is directly attached to an individual finger’s joint, enabling the rotation of a finger phalanx. While this structure ensures the highest level of accuracy in grasp and release motions, it often results in a gripper that is heavy and bulky. To address these limitations, many gripper designs incorporate transmission links to drive the finger joints.

Pneumatic and hydraulic: These types of actuators are extensively used in both conventional and soft grippers. In such cases, air pressure or liquid pressure is employed to create motions in the gripper for the purpose of grasping or releasing objects. This approach is commonly employed in cylinders, Pneunet mechanisms, jamming-based systems, vacuum actuators, and more. Fluid actuators offer advantages such as rapid actuation, hygienic operation, and compactness. However, achieving precise control and minimizing noise in gripper movements can be challenging with fluid-based actuators.

C-D: This type of actuation is commonly used to control the fingers with rigid phalanges. In this mechanism, two ends of a cable are anchored to the fingertips, while the middle section is affixed to a pulley connected to a motor shaft. When the motor rotates, it pulls one branch of the cable while releasing the other, resulting in the movement of the joint or finger in a specific direction. Actuators offer benefits such as reducing the gripper’s inertia during operation by placing the motor away from the fingers. Additionally, the tension along the cable remains constant throughout its length, ensuring a consistent grasping force.

SMAs: Actuators are used in robotic grippers to control the joints of the fingers. They offer advantages such as a low weight-to-force ratio, minimal noise, and a compact size. The design of SMAs allows for a seamless transition between grasping and releasing states through thermal heating [268]. This mechanism enables the passive holding of objects even when the power to the gripper is cut off.

Electro-adhesive: Electro-adhesive actuators operate by applying power to electrodes embedded in a dielectric substrate. The presence of opposite charges between the electro-sticky pads and the substrate creates adhesive forces that enable the gripper to achieve a secure grip on objects.

Other types of actuators: Various other types of actuators have also been employed in gripper designs, expanding beyond the methods mentioned earlier. Examples include shape-adaptive magnetorheological elastomers and electroactive polymer actuation. These alternative actuators provide additional options for creating innovative and effective gripper mechanisms.

### 5.2. Applications

B-I soft and rigid grippers have found a multitude of applications across various industries and domains, owing to their ability to mimic natural mechanisms and adapt to different objects and environments. These grippers combine the advantages of biological systems with advanced robotic technology, enabling them to excel in tasks that were once challenging for traditional grippers.

#### 5.2.1. Applications of B-I Soft Grippers

Soft grippers have shown promise in medical applications such as minimally invasive surgeries (the Da Vinci Surgical System), endoscopic procedures, and drug delivery. Their compliant nature allows them to interact with delicate tissues and perform intricate tasks without causing damage. In research laboratories, these grippers are used for handling delicate samples, pipetting, and micromanipulation. Their softness and flexibility enable the accurate and controlled handling of sensitive materials. Soft grippers are ideal for handling fragile and irregularly shaped objects like fruits, vegetables, and baked goods. Their adaptability ensures gentle handling, reducing product damage and waste during harvesting, sorting, and packaging processes. In addition, these grippers equipped with tactile sensors assist people with disabilities in daily activities like grabbing objects or turning knobs. Soft robotic grippers have found significant applications in warehouse automation to enhance efficiency in picking and sorting tasks. Suction-based soft robotics grippers are used for box handling and parcel sorting in distribution centers. On the other hand, jamming-based soft robotic grippers are used in bin picking and for handling delicate items like electronics or glassware.

#### 5.2.2. Applications of B-I Rigid Grippers

Rigid grippers with anthropomorphic designs are employed in industrial manufacturing and assembly lines for tasks such as picking, placing, and manipulating components in precise positions [269]. These grippers are also used in construction and maintenance tasks that require heavy lifting, such as moving construction materials or repairing large structures. Moreover, rigid grippers on rovers like the Mars Curiosity Rover manipulate tools and collect samples from the Martian surface. Finally, the grippers are also used in scientific research and exploration, such as underwater studies, archaeological excavations, and studying inaccessible environments.

Both soft and rigid B-I grippers continue to evolve, opening doors to new applications and use cases across industries. Their ability to adapt, conform, and interact with objects in ways similar to natural organisms makes them powerful tools for advancing automation, efficiency, and safety in various fields.

## 6. Conclusions

The field of gripper technology has witnessed remarkable advancements through the integration of B-I designs, encompassing both rigid and soft gripper systems. The influence of natural mechanisms and structures from animals and plants has driven innovation in gripper development, leading to the creation of highly efficient and adaptable robotic manipulators.

Rigid grippers, drawing inspiration from the bio-mechanics of human and animal limbs, have paved the way for highly capable and versatile robotic systems. These grippers, with their anthropomorphic configurations and intricate joint arrangements, have found applications in industrial automation, manufacturing, and precise manipulation tasks. By mimicking the dexterity and precision of human hands, these grippers have transformed industries by enhancing productivity and automation capabilities.

On the other hand, soft grippers, inspired by the compliant nature of biological organisms, have introduced a paradigm shift in manipulation tasks. The utilization of flexible materials and mechanisms has enabled these grippers to adapt to a wide range of object shapes and sizes, making them ideal for delicate and complex tasks. The integration of SMPs, low-melting-point alloys, and other novel materials has led to the development of grippers with variable stiffness, further enhancing their ability to interact with different objects and environments.

The synergy between B-I concepts and robotics has not only expanded the horizons of gripper technology but has also bridged the gap between the artificial and natural worlds. The diverse applications of these grippers, ranging from industrial automation to healthcare and beyond, underscore their transformative potential across various domains. The continuous exploration of B-I principles holds the promise of unlocking even more sophisticated and efficient gripper systems, shaping the future of robotics and manipulation.

In conclusion, the convergence of biology and robotics in gripper design has yielded systems that emulate the capabilities of living organisms, while also driving technological progress. This research paper explored the rich landscape of rigid and soft B-I grippers, highlighting their design principles, mechanisms, and applications. As research in this field continues to evolve, it is anticipated that these B-I grippers will play an increasingly significant role in revolutionizing the way we interact with and manipulate the world around us.

## Data Availability

Data sharing not applicable.
